# Peer Mentoring by Medical Students for Medical Students: A Scoping Review

**DOI:** 10.1007/s40670-024-02108-7

**Published:** 2024-07-04

**Authors:** Christos Preovolos, Abby Grant, Morgan Rayner, Kylie Fitzgerald, Louisa Ng

**Affiliations:** 1 Melbourne Medical School, The University of Melbourne, Melbourne, Australia; 2 Department of Rehabilitation Medicine, The Royal Melbourne Hospital, Melbourne, Australia

**Keywords:** Medical students, Peer, Near-peer, Mentoring

## Abstract

Medical school transitions pose challenges for students. Mentoring programs may aid students, but evidence supporting peer/near-peer mentoring in medical school is unclear. Our review explores peer mentoring’s benefits, elements for success and challenges. Searches in major databases yielded 1676 records, resulting in 20 eligible studies involving 4591 participants. Longitudinal (*n* = 15) and shorter, focused programs were examined. Mentors and mentees reported psychosocial, professional and academic benefits. Essential elements included matching, orientation and clear goals, with training crucial yet balanced to avoid mentor overload. Social congruence underpinned successful peer mentoring, particularly benefiting under-represented groups. Challenges include balancing mentor load and logistics.

## Introduction

Higher education is a life-changing time for students, often young adults, who are developing their sense of self, role in society, independence and career trajectories. Certain groups, such as First Nations students [1], international students [2] and those from lower socioeconomic backgrounds [3], are more likely to face additional challenges during this transition period.

In addition to the issues which arise during life transitions, medical training brings a unique set of challenges, which include high academic expectations, rigid course structures and exposure to a complex, sometimes distressing, clinical environment [4, 5]. As such, medical students may experience a negative impact on well-being, interpersonal relationships, quality of life and academic performance during their degree [4, 6–8], especially when transitioning from pre-clinical into clinical years [9, 10].

One possible method to provide additional support for medical students is through mentoring programs [11–14]. In such programs, relationships are typically established between more experienced individuals (‘mentors’), and those more junior (‘mentees’), with the aim of promoting personal and professional development, as well as psychosocial support [8, 15]. Within medical schools, mentors for medical students can be faculty, senior or junior clinicians, or other students. Mentor selection may include factors such as academic performance, specialty, or sociodemographic backgrounds (e.g. cultural or religious [16, 17]). Whilst faculty and clinician mentors bring more experience, peer mentors are typically considered more available and approachable [18, 19]. Peer mentors may be peers at the same level in their training, or near-peers, who have progressed academically beyond the mentee. In this review, we use the term peer mentoring to include both peers and near-peers for conciseness.

Peer mentoring programs, especially where both mentors and mentees are medical students, are thought to benefit from the high level of ‘cognitive and social congruence’ that exists between students in similar educational environments [20]. The shared understanding of academic frameworks, knowledge and interpersonal roles allows for the development of complex and comfortable mentor–mentee relationships [8, 20]. Furthermore, elements exclusive to student mentoring, such as the use of familiar language, similar social roles and an empathic understanding of the ‘student experience’, may provide benefits that faculty mentoring does not. A diverse student cohort, moreover, means that social congruence can extend to students from culturally and linguistically diverse backgrounds [20]. Accordingly, one review found that the ‘comfortable’ learning environment established by peers has been highlighted as an important advantage over formalised teaching [20]. Similar principles apply for ‘peer-assisted learning’ (or peer teaching). However, the difference is peer-assisted learning primarily targets academic goals, whilst peer mentoring prioritises wellbeing and support [8, 11, 15, 21, 22].

Whilst mentoring in the medical profession is relatively common, there is also evidence to support mentoring of medical students. A 2020 qualitative study evaluating different university initiatives to reduce stress found that peer mentoring ‘effectively reduce(d) stress in medical students, and facilitate(d) their transition into medical school’ [11]. A 2018 systematic review [15] included five studies on near-peer mentoring programs, focusing on outcomes for first-year health professional (medical, allied health and nursing) students and reported personal development and psychosocial benefits for these mentees. In 2019, a large narrative review [23] with 82 included studies on medical student mentoring also reported similar findings. This study showed mentees benefited from attainment of skills, knowledge and personal/professional development, whilst mentors improved in leadership and communication skills. However, the mentors in these studies were predominantly junior doctors, not medical students. Another systematic review [24] focusing on the impact of mentoring on medical students from under-represented minority groups identified benefits for academic, research and career pathways. Finally, a systematic review [22] on peer mentoring, specifically in Iranian medical schools, found benefits for both mentees and mentors.

Though the evidence for peer mentoring programs for medical students is promising, and several systematic reviews have been conducted to evaluate the utility of mentoring for medical students, none to date have specifically reviewed the benefits of near-peer mentoring by medical students, for medical students. This scoping review aims to determine the benefits and challenges of medical student peer mentoring programs, and identify elements for program success, accounting for differences in curriculum, program structure and mentor/mentee composition. Findings may inform future research and discourse and assist medical schools in the organisation and optimisation of their peer mentoring programs to benefit their students.

## Methods

The scoping review was conducted in accordance with the Joanna Briggs Institute methodology for scoping reviews [25], and registered on the 6th of August 2023 with the Center for Open Science, 10.17605/OSF.IO/XEA63. The scoping review methodology was selected to map existing literature, both published and unpublished and to determine gaps with the aim of informing future research.

### Search Strategy

An initial limited search of Ovid MEDLINE, EMBASE and ERIC was undertaken using a search strategy developed with the assistance of a librarian. The text words contained in the titles and abstracts of relevant articles and the index terms used to describe the articles were used to develop a full search strategy with further librarian assistance. As ‘mentoring’ is not a MeSH term, and various phrasing is used to describe mentoring programs, the two-step development of the final search strategy was required. The following databases were searched: Medline, ERIC, EMBASE, Web of Science, WorldWideScience and the British Library on 22/1/2024. Keywords and MeSH terms used included medical student*, peer mentor* and medical education. Boolean operators (AND, OR, NOT) were used to refine search results. The search strategy was then adapted for other databases (see Appendix). The reference list of included studies and relevant reviews were screened for additional studies. Grey literature was searched using OpenGrey (http://www.opengrey.eu) and GreyNet (https://www.greynet.org/).

### Inclusion and Exclusion Criteria

Inclusion criteria were as follows:

- Primary studies of any design published in English.
- Participants: Medical students undertaking a primary medical degree (undergraduate or postgraduate)—applicable to both mentor and mentee participants.
- Intervention: Peer or near-peer mentoring programs.
- Comparison: No program or different types of mentoring programs.
- Outcomes: Any that related to mentors or mentees including satisfaction with program, academic skills, professional skills and health and wellbeing, elements for peer mentoring program success and challenges of these programs.

Exclusion criteria were as follows:

- Doctors as mentors.
- Peer-assisted teaching and learning programs.
- Conference papers, abstracts and opinion pieces.

**Fig. 1:**
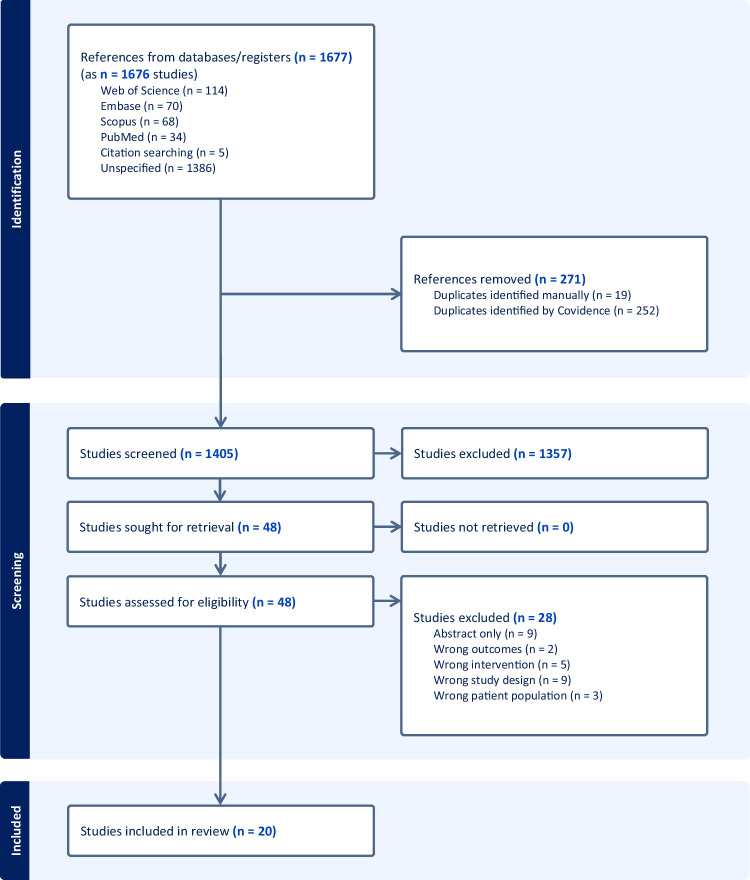
PRISMA flowchart outlining search results, screening and study inclusion process

Two reviewers (AG/MK/CP and MR) independently screened titles and abstracts obtained from the search, excluding irrelevant citations, using Covidence. Full-text articles were subsequently retrieved and reviewed for final inclusion. Disagreements were discussed amongst three reviewers (AG/MK/CP, MR and LN) and resolved through consensus. The selection process was reported via Preferred Reporting Items for Systematic Reviews and Meta-Analyses (PRISMA) flow diagram (Fig. 1).

### Data Extraction

Data were extracted by one reviewer (AG/MK/CP) and checked by a second (LN/MR). The following data were extracted:

- Study design and country.
- Participants: numbers of mentors and mentees, year of study in medical school.
- Interventions: program description, process of matching of mentees to mentors, duration, support provided for the program.
- Measurements, assessment time points and outcomes such as benefits, elements contributing to program success and challenges.

The heterogeneity of the studies and variation in outcome measures (most of which had been self-developed) meant that pooling results for a meta-analysis was not possible.

### Reflexive Statement

The research question was ‘What are the benefits and challenges of medical student peer mentoring programs and what are the elements associated with their success?’ This question was derived from the authors’ experience as medical educators and medical students, namely, the challenge to develop and optimise a peer mentoring program for medical students which would best support their psychosocial, professional and academic needs. Therefore, the search strategy was developed in light of the authors’ joint interests. The two authors who primarily undertook the data extraction (AB, CP) were medical students at the time and participants of a peer mentoring program in their clinical school, and the extraction was verified by two other authors (MR, LN), one of whom (LN) had developed the clinical school mentoring program. The inclusion of a non-medical practitioner who had not been directly involved in the peer mentoring program (KF) ensured a level of rigour in data analysis and interpretation.

## Results

### Overview

A total of 1676 citations were identified through the initial search (Fig. 1). Of these, 48 full-text studies were assessed for eligibility and 20 (two of the same program) [13, 26] met the inclusion criteria. Of the included studies, seven were cohort studies [13, 14, 17–19, 27, 28], seven mixed-methods [26, 29–34], three qualitative [16, 35, 36] and three cross-sectional [37–39] (Table 1). There was a total of 4591 participants (*n* = 533 mentors; *n* = 1625 mentees; *n* = 2878 unspecified) with a range of 9–2362 participants in each study. When including only studies which specified year level of mentees/mentors, most mentees were in their first clinical year (*n* = 898), whilst most mentors were near peers in either their ultimate or their penultimate year (*n* = 224). Three programs had mentors and mentees from the same year level [30, 33, 34]. In general, mentors and mentees were randomly matched, typically following a selection process for mentors. Six [14, 17, 27, 30, 37, 38] of the included studies considered specific characteristics (such as international students, being taught by the same faculty, personal relationships) when matching mentors and mentees. Mentoring groups were generally small, with one notable exception where a social media platform was used for large group mentoring (> 1000 students within the same year level) [33]. Twelve studies reported a component of peer teaching as part of the mentoring program [13, 14, 16, 26, 28–31, 36].

**Table 1:** Summary of included studies

Study (Country)	Design	Participants	Intervention	Assessment time points	Measure	Outcome
Longitudinal programs						
Abdolalizadeh et al. 2017 [35] (Iran)	Qualitative	Mentors—fourth year medical students (clinical, *n* = 6; non-clinical, *n* = 6) Mentees—first year medical students randomly selected from cohort (n = 36)	**Recruitment of mentors**: volunteers **Allocation of mentees to mentors**: N/A; 2 mentors (one clinical, one preclinical) and 6 mentees per group **Contact logistics**: Contact made via telephone calls, email and face-to-face in varying frequency **Training and orientation provided**: Yes, were trained on purpose of mentoring and communication skill development **Activities organised**: Weekly inter-mentor sessions for idea sharing **Duration of program**: Academic year	Conclusion of academic year	Four focus groups administered to mentors and mentees Questions were regarding experiences in the program, perceptions of development, strengths and weaknesses of the program, and the quality of each component Length of each focus group was ~ 60 min. All meetings were recorded and transcribed **Data analysis**: Conventional content analysis	• Mentors and mentees agreed on several characteristics that made a good mentor: patience, active contact and persistence • Mentees commented that their mentor played a role in their academic progress, mainly via discussions regarding study methods/techniques • Mentees benefitted from having both clinical and non-clinical mentors • The perceived independence of the program from the medical school was seen as a bonus for mentees and mentors • Diversity in communication methods was seen as a strength of the program, however most participants preferred face-to-face meetings • Mentors reported both professional and social development
Altonji et al. 2019 [27] (USA)	Cohort study	Mentors—2nd year medical students (*n* = 112) Mentees—1st year medical students (*n* = 190)	**Recruitment of mentors**: Volunteers **Allocation of mentees to mentors**: Pairing done by student leaders (learning representatives in their second year, elected by their peers), no guidelines on pairing but was guided by personal relationships the leaders had to both mentors and mentees **Contact logistics**: Casual meetings at least once a month **Training and orientation provided**: Yes, large group training received once **Activities organised**: None **Duration of program**: Academic year	Conclusion of academic year	One survey and one focus group administered to mentees Mentorship Effectiveness Scale (MES)—twelve Liker-scale items to assess effective mentor behaviours, including perceptions of the benefits of the program, overall satisfaction and accessibility of contact of the mentor to the mentee A focus group was used to modify the survey to suit the needs of the study, and was non-evaluative (*n* = 9, first years) **Data analysis**: Stepwise linear regression	• Items that scored higher than 3.5 on the MES included the following: mentor was accessible (4.2 ± 0.99), approachable (4.41 ± 1.16), supportive and encouraging (4.36 ± 1.08), answered questions satisfactorily (4.13 ± 1.19), and suggested appropriate resources (4.09 ± 1.21), demonstrated expertise in an area of need (3.85 ± 1.16), provided useful and constructive critique (3.68 ± 1.43), provided guidance on how to succeed in medical school (3.83 ± 1.41), and provided useful advice on how to study (3.84 ± 1.26) • Overall satisfaction with the mentoring program was high (7.47 ± 2.45, out of 10) • Predictors to perceived satisfaction and benefit included the following: mentor knowledge, mentor advice on handling medical school, mentor providing guidance on professional matters, there was no statistically significant benefit to either male or female mentors; however, regular meetings and involvement were seen as a positive predictor • Results of focus group were used to modify the survey only and hence not separately reported
Andre et al. 2017 [29] (USA)	Mixed methods	Mentors—fourth year medical students (‘MiMs’) (*n* = ~ 60) Mentees—first, second and third year medical students participating in the ‘Veritas’ mentoring program (*n* = ~ 220/year level)	**Recruitment of mentors**: Volunteer **Allocation of mentees to mentors**: All students randomly assigned to one of 20 Veritas groups upon enrolment; each group consisted of 11 students from MS1-MS4. 3 volunteer mentors allocated to each ‘Veritas’ group randomly **Contact logistics**: Group and one-on-one meetings, minimum once a month **Training and orientation provided**: None **Activities organised**: Weekly mentor meetings with Veritas director **Duration of program**: Academic year	Conclusion of academic year with each new cohort	Two self-developed surveys (piloted and refined prior to use) measuring effectiveness of peer-mentoring and perception of improved experience of medical school, administered to mentors and mentees Survey 1 (Veritas All Students Survey) asked mentees Likert scale questions regarding MiMs engagement with getting to know their mentees, assisting with rotation planning, stress management, guidance and advice, emotional support, the mentees preference for approaching MiMs over faculty mentors, and if the mentees saw MiMs a useful resource Survey 2 (Mentors in Medicine, or MiMs survey) asked the mentors questions about their perceived preparation in running meetings, providing guidance, personal and professional development, and their perceived success in their mentoring role **Data analysis**: Mantel–Haenszel Chi square	• The findings showed that in general, peer mentors were able to provide advice about academic planning, psychosocial support, promoted interpersonal relationships, peer support and a safe space for mentees to discuss personal issues • Level of satisfaction varied year to year • In 2012 and 2013, 100% of mentors said they would volunteer again the following year • 71% of mentors felt sufficiently prepared in their role • Among first- and second-year mentees, there was an overall perceived benefit in performance year to year from 2011 to 2014 • Over the years there is a visible trend in improvement in satisfaction with the medical school experience via post hoc testing • Other up trending components of the survey included: discussion of professionalism questions, academic planning, future year planning, networking, peer support, relationships between classes, social supports and discussions of emotional issues that relate to the patient experience
Behkam et al. 2022 [37] (Iran)	Cross-sectional	Mentors: third/fourth year medical students Mentees: first year medical students Non-mentees: first year medical students	**Recruitment of mentors**: Volunteers **Allocation of mentees to mentors**: Assigned based on gender, ethnicity, living place, scientific background. One-to-one allocation **Contact logistics**: Face to face meetings on a weekly basis **Training and orientation provided**: 3 day workshop on concept of mentoring, communication, program rules/expectations **Activities organised**: Weekly sessions centred around assisting new students with transition to medicine and aimed at improving their perception of educational environment **Duration of program**: Academic year	During first year (between March and September 2019)	DREEM (Dundee Ready Education Environment Measure) tool (Persian translation) used to assess students’ overall perception of their learning environment Student responses were compared across the mentored and non-mentored groups and tested for significant differences **Data analysis:** *T*-test, ANOVA to compare means of quantitative variables. *P* < 0.01 used for statistical significance	• 169 out of 190 study recruits (87 mentees, and 82 non-mentees) participated in data collection • Total DREEM score not different based on demographics • Mean DREEM scores of students with mentors not statistically different from those without • No DREEM item was significantly different across the two groups • More mentees stated that ‘there is a good support system for students that get stressed’ than non-mentees, *P* = 0.009
Chatterton et al. 2018 [17] (UK)	Cohort study	Mentors—selected second and third year medical students (*n* = 49) Mentees—first year medical students	**Recruitment of mentors:** Initial year organised by Senior Tutors in collaboration with two student volunteers. Subsequent years was carried out by group of experienced peer mentors from the previous year **Allocation of mentees to mentors:** Certain groups matched accordingly (e.g. international student with international student peer mentor) **Contact logistics:** Peer mentors encouraged mentees to make contact when required, and contact made by peer mentors at various ‘high-stress’ points **Training and orientation provided:** 3 training sessions in total amounting to 6 h formal training over an 8-month period **Activities organised:** Informal welcome event **Duration of program:** Academic year	Post second and third training session (mentors)	Five-point Likert scale questionnaire evaluating peer mentor’s perception of whether the training sessions met their expectations, application of knowledge learned, confidence in their roles, perceived support, knowledge about where to sign-post mentees when indicated and overall questions about the quality of the trainers and materials/course content Post-third session: similar to post-second training session questionnaire, but was amended to incorporate questions eliciting specific feedback on new issues discussed and not covered in former session (resilience, mental health and revision/ study skills)	• Both sessions were rated highly with a mean (± standard deviation) session of 4.37 (± 0.21) after the second training session from 29 responses, and 4.33 (± 0.38) following the third session from 32 responses • Following the second session, the question rated highest was ‘I feel that I will be supported in my role as peer mentor’ with a mean score of 4.72 and the question rated lowest was ‘the training met my expectations’ with a mean score of 4.03 • Following the third session, the question rated the highest was ‘The trainer was knowledgeable’ with a mean score of 4.72, and the question rated the lowest was ‘My own revision strategy will change in light of the station on revision/study skills’ with a mean score of 3.13
Cho and Lee 2021 [30] (Korea)	Mixed methods study	Mentors—either in the same year level as their mentee, or two academic years above Mentees—preclinical second year (PY2), first year (Y1) or second year (Y2) medical students Total *n* = 67 participating in peer-mentoring program; breakdown unspecified	**Recruitment of mentors:** Volunteer **Allocation of mentees to mentors:** Choice to pair themselves or school to pair mentor and mentee **Contact logistics:** Meeting approximately 4 or more times per semester **Training and orientation provided:** At least 2 h of education on mentoring, rules to follow and appropriate communication and feedback skills **Activities organised:** None **Duration of program:** 2 years	Conclusion of program	Quantitative: survey assessing general information regarding program implementation (how often students participated; how mentors and mentees would meet; how many times, where, and at what times mentors and mentees would meet) and appropriateness (satisfaction with the program, purpose for participation and mentors’ role). Program satisfaction and appropriateness were answered using the Likert scale of 1–5 (with 1 being ‘not at all’ and 5 being ‘very high’) Qualitative: open-ended survey questions (regarding benefits and outcomes gained by both) and focus group interviews (1–2 h of probing questions: (1) why and how students participated in the mentoring program, as well as how they felt or what they thought about the program; (2) the personal experiences of students; and (3) the significance of their participation. Also included discussion regarding what more was needed to improve) **Data analysis:** Mann–Whitney *U*-test was used to compare differences in continuous variables between groups due to small sample size	• Program implementation: 84% face-to-face meetings, 74% used text messages, 64% had meetings for 1–2 h, 21% had meetings for less than an hour • Participant satisfaction and evaluation of program: mentors rated 3.6 (± 0.5) and mentees rated 3.8 (± 1.0) • Purpose of students’ voluntary participation and role of mentors: most common answer for both mentors and mentees were ‘teaching assistance for test-taking strategies and guidelines’ followed by emotional support • Qualitative outcome and mutual benefits: improved grades for mentees’, an increased willingness and motivation to study, improved lifestyle patterns, emotional support. Specific to mentees’: improving interpersonal relationships and obtaining support. Specific to mentors: better understanding of previously learnt content, sense of responsibility • Qualitative negative experiences: one mentee response ‘feeling pressure’, and mentors ‘not improving mentees’ learning habits or attitudes nevertheless continuous asking improvement’, ‘not doing homework’ and ‘not contacting or requesting in advance’ • Suggestions for improvement from both mentees and mentors: ‘specific guidelines on how to do’, ‘training and education for mentees’ and ‘appropriate matching between mentor and mentee’. Mentors specifically suggest knowing the characteristics and tendencies of both mentee and mentor, the mentee must have a clear purpose when they participate, and the mentee’s voluntary participation is essential
Fleischman et al. 2019 [38] (USA)	Cross-sectional study	Mentors—77 fourth year medical students in the formal mentoring program (‘Cicerone’) Mentees—first and second year medical students; number not specified	**Recruitment of mentors:** Not specified **Allocation of mentees to mentors:** Defined by programs that ‘formally and thoughtfully assign mentor to mentee’. 4th year medical students assigned to 2nd years, and 3rd year students to 1st years **Contact logistics:** Not specified **Training and orientation provided:** Not specified **Activities organised:** Not specified **Duration of program:** Not specified	Conclusion of academic year	Survey including categorical options and open-ended questions. Respondents who participated in Cicerone peer mentoring program were specifically compared to those who did not participate in any formal mentoring A second purely descriptive analysis was performed including only those who did not participate in any mentoring to understand their motivation for not participating A third analysis of only Cicerone (formal peer mentoring) to assess their experience **Data analysis:** Simple statistics used, including Fishers exact and chi-squares for categorical variables, and t tests and Kruskal–Wallis for continuous variables	• 60% response rate (107/178); 42 participated in Cicerone program (77% response rate), 17 mentored in other programs, and 48 non-mentors responded • Non-mentors did not participate in the mentoring program due to: not knowing about program (69%), not having time (26^) and not believing it was useful (23%). Men were more likely than women to cite ‘not knowing about the program’ and ‘did not believe it would be useful’ • Cicerone mentors were twice as likely to believe being a mentee was beneficial in medical school. 75% of mentors felt it was a worthwhile experience • Mentors were 4 times more likely to believe that being a mentor in an SSM program was valuable and 83% stated they would mentor again • Career choice was discussed by 79% of pairs, and 42% mentors felt more positively about their career choice
Nebhinani et al. 2020 [19] (India)	Cohort study	Mentors—undergraduate medical students in their 3rd, 4th and first half of 5th year, or faculty members Mentees—undergraduate first and second year medical students (*n* = 162)	**Recruitment of mentors:** Volunteer **Allocation of mentees to mentors:** Either peer or faculty mentor randomly assigned to 2–4 mentees **Contact logistics:** Encouraged at least one mentorship meeting every fortnight **Training and orientation provided:** Guidelines for mentees and mentors **Activities organised:** Discussions **Duration of program:** 8 months	Not specified; questionnaire distributed to ‘receive feedback for ongoing mentorship program’	Semi-structured questionnaires, which involved survey items and questions assessing perceived benefits and qualities of mentors, barriers in mentorship meetings, commonly discussed topics in mentorship meetings, and the role of faculty mentor and their relationship **Data analysis:** Comparison between the groups have been done using chi-square test and *t*-tests for categorical and continuous variables respectively	• Over the 8-month period, mean number of meetings were 4 with faculty mentor and 11 with peer mentor • For both the faculty member and peer mentors, mentees commonly reported that their qualities included being understanding, being supportive and having a helping attitude • The most common barriers faced with faculty members include difficulty synchronising meeting times, students’ lack of time or no interest from students • The most common barriers faced with peer mentors include difficulty synchronising meeting times, mentors’ lack of time and then students’ lack of time Commonly discussed topics with both faculty and peer mentors include education in general, life as a medical student and work-like balance related issues
*Neufeld et al. 2020 [13] (Canada)	Cohort study	Mentors—second year medical students as ‘instructor-mentors’ *n* = 17 Mentees—first year medical students as ‘learner-mentees’ *n* = 38	**Recruitment of mentors:** Voluntary **Allocation of mentees to mentors:** 2 s year mentors matched with 2 or more first year students weekly **Contact logistics:** Weekly from October to April with exception for holidays and exams **Training and orientation provided:** Mentors received only general instructions prior to mentoring without any specific prerequisite training **Activities organised:** ‘PULSE’ (Peers United in Leadership and Skills Enhancement) sessions; topics coincided with students’ curriculum and progressed from basic history taking and physical exams, to procedural (e.g. otoscope) and interpretative skills (e.g. chest x-ray), to problem solving (e.g. formulating differentials, investigations and management plans) and presenting cases **Duration of program**: 6 months	Within 3 months (April–June) post-PULSE (Peers United in Leadership & Skills Enhancement) sessions	General feedback form, and adapted 6-item Learning Climate Questionnaire which measures perceived autonomy support	• First year medical students attended an average of 2–3 sessions • PULSE helped reduce performance anxiety around OSCE’s, not only providing ‘a safe and relaxing learning environment, but comradery and professional relationships as well’ • Mentors ‘were incredibly helpful and had a different perspective than instructors, which made it easier to ask questions’ and ‘supportive feedback and non-judgmental environment’
*Neufeld et al. 2021 [26] (Canada)	Mixed methods study	Mentors—second year medical students as ‘instructor-mentors’ *n* = 37 Mentees—first year medical students as ‘learner-mentees’ *n* = 53	**Recruitment of mentors:** Voluntary **Allocation of mentees to mentors:** 2 s year mentors matched with 2 or more first year students weekly **Contact logistics:** Weekly from October to April with exception for holidays and exams **Training and orientation provided:** Mentors received only general instructions prior to mentoring without any specific prerequisite training **Activities organised:** ‘PULSE’ (Peers United in Leadership and Skills Enhancement) sessions; topics coincided with students’ curriculum and progressed from basic history taking and physical exams, to procedural (e.g. otoscope) and interpretative skills (e.g. chest x-ray), to problem-solving (e.g. formulating differentials, investigations and management plans) and presenting cases **Duration of program**: 6 months	Conclusion of academic year	3 validated Likert-type surveys: • **LCQ** (Learning Climate Questionnaire): 6-item scale measuring learners’ perspective of the autonomy supportiveness of their mentors • **W-BNS** (Basic Need Satisfaction at Work Scale): 21-item scale measuring workplace need satisfaction • **PCS** (Perceived Competence Scale): 4-item scale assessing feelings of competence in a given task, referring to learning or teaching clinical skills in PULSE sessions **Data analysis:** Cronbach’s alpha values for each scale to calculate reliability Linear regression used to assess learner perception of how PULSE’s learning climate impacted need satisfaction and how need satisfaction impacted perceived competence in learning. Further analysis of how each of the autonomy, competence and relatedness satisfaction subscales predicted their perceived competence in learning as separate independent variables Linear regressions used to assess mentor perceptions of how overall need satisfaction in PULSE impacted perceived competence in teaching, followed by how need satisfaction subscales predicted perceived competence in teaching	• Both groups’ mean scores for autonomy, competence and relatedness supported high levels of need satisfaction • PULSE’s learning climate positively related to learners’ overall need satisfaction (*R*^2^ = .39, *p* = .01) • When overall need satisfaction was entered into the regression as the independent variable, it positively related to perceived competence in learning the material (*R*^2^ = .32, *p* < .05) • Mentors’ overall need satisfaction in PULSE positively related to their perceived competence about teaching the material • Both mentors and mentees found PULSE sessions highly supportive to their autonomy, competence and relatedness; this corresponded with greater perceived competence about learning and teaching of clinical material
Shafiaai et al. 2020 [31] (Malaysia)	Mixed methods study	Mentors—‘senior year medical students’ (*n* = unspecified) Mentees—undergraduate second year pre-clinical medical students	**Recruitment of mentors:** Elected through an interview for their excellent communication and inter-personal skills and have achieved excellent results in all course-related examinations with a ranking of upper third quartile in their cohort of students **Allocation of mentees to mentors:** Not specified **Contact logistics:** Weekly basis **Training and orientation provided:** Two-day Peer-Assisted Study Sessions (PASS) leaders training is conducted by a certified trainer-supervisor for PASS program **Activities organised:** 1 hourly weekly based integrated learning session through problem-solving and active discussion regarding various topics anatomy, biochemistry, physiology and clinical skills, etc **Duration of program:** One 12-week semester	Baseline and at the end of 12-week program (questionnaire) Focus group discussion 1 year after completion of 12-week program	Validated questionnaire (5-point Likert scale) which included 12 items with focus on establishing level of communication skill and pedagogical skill Structured focus group interview exploring whether or not participation in the PASS program as peer leaders helped in developing skills required to become a successful doctor in the future from participant’s point of view	• On completion of PASS program, peer leaders reported an increase in oral and written skills to engage with junior students, and skills to develop interaction and collaboration • Peer leaders reported a significant increase in leadership skills and teamwork, stress and time management skills, interpersonal and critical thinking skills, ability to create an effective learning environment, ability to learn new skills, problem-solving and innovative thinking, facilitate teaching sessions and ability to develop independent learning amongst their students • Qualitative analysis revealed 2 main themes: personal growth and professional growth • Within the theme of Personal Growth, 4 skill subthemes were identified: communication, leadership, learning and pedagogical skills Within the theme of Professional Growth, 4 subthemes were identified: administrative, experience, responsibility and time management
Singh et al. 2014 [18] (India)	Cohort study	**Mentors**—a mix of second, third, and fourth year volunteer medical students (*n* = 57). Plus, faculty supervisors (*n* = 52) **Mentees**—first year medical students (*n* = 148)	**Recruitment of mentors**: Volunteers **Allocation of mentees to mentors:** Randomly to 52 groups; one mentor and one faculty supervisor for to three mentees **Contact logistics:** Meetings, frequency not specified, method of contact not specified **Training and orientation provided:** None **Activities organised**: 2 ‘open house’ meetings with entire mentoring cohort **Duration of program:** Academic year	Conclusion of academic year	Two self-developed questionnaires were administered to mentors and mentees Questionnaire 1: to mentees, questions focused on the amount and quality of the contact with mentors Questionnaire 2: to mentors, focused on what they gained from the program, and the barriers that affected them **Data analysis**: Chi-square for categorical variables, student unpaired *t*-test for continuous variables	• Average contact time between peer mentors and mentees increased when compared with contact time between mentees and faculty mentors • Peer mentors reported benefits to them from engagement in the program; particularly improvement in problem-solving, responsibility, confidence and counselling, dual-way mentoring
Taylor et al. 2013 [14] (USA)	Cohort study	Mentors—second year medical students, in a ‘teaching academy fellow’ role (*n* = 28) Mentees—first year medical students (*n* = 96)	**Recruitment of mentors**: Volunteers **Allocation of mentees to mentors**: One mentor to four mentees; mentors assigned to groups taught by the same faculty as in their first year when possible **Contact logistics**: Meetings twice a week **Training and orientation provided**: Yes, 2-h orientation **Activities organised**: Group discussions, wellbeing checks **Duration of program**: Academic year	Academic years ending mid 2009 mid 2010 Immediately post orientation (mentors) 2-months post orientation workshop (mentors) Conclusion of academic year (mentees)	Non-validated surveys administered to mentors and mentees The first after the orientation workshop received by the mentors, and then 2 months into the program. Surveys aimed to examine the application of skills taught to mentors in orientation workshops, as well as student satisfaction as mentors **Data analysis**: Not specified	• Evaluation of orientation: Reported increase in confidence in ability to provide adequate and constructive feedback (*p* < .001) • Evaluation of whole academic year program: 94% of TA mentors suggested the orientation workshop be continued in years to come, with suggestions on how to improve made • Majority of mentees were satisfied with the quality of feedback, reported confidence in performing medical interview increased after mentoring sessions
Yang et al. 2021 [36] (USA)	Qualitative	Mentors—third and fourth year medical students randomised to Education Centered Medical Home (ECMH—a team-based clerkship program consisting of students across all 4 year levels) Non-mentors: third year students randomised to the Individual Preceptorship stream (IP; one-on-one clerkship program pairing a student with a physician) Mentees—students from all four year levels participating in the ECMH program	**Recruitment of mentors:** Students randomly assigned to ECMH stream **Allocation of mentees to mentors**: students randomly assigned to the ECMH stream. Group size not specified **Contact logistics:** Students attended ECMH clinic 26 times throughout third and fourth years **Training and orientation provided:** None **Activities organised**: None **Duration of program:** 4 years	3 months into the semester of third year	One-to-one semi-structured interview with M3 mentors using an interpretivist paradigm. Interview questions included: What opportunities have you had to teach your peers, what opportunities have you had for your peers to teach you, can you tell me about a time when you have served as a role model or mentor for other medical students and in general, how does it feel to be an M3 in clinic now, thinking back to when you were an M1? **Data analysis:** two cycle-based inductive approach to coding and analysis	• Derived three main themes: (1) diversity of peer teaching and mentoring opportunities, (2) transitioning one’s role from learner to teacher and (3) personal and professional development • Whilst participants from both clerkships participated in peer teaching and mentoring experiences, ECMH students described more opportunities to interact with students across all years of medical school training, noting that ‘getting that guidance and in turn being able to teach is a valuable experience’ • ECMH students further perceived the responsibility of creating a comfortable learning environment for others • Students from both clerkships (ECMH and IP) reflected on ‘learning through teaching’, that teaching served as a reaffirmation of the knowledge they gained, and that teaching experience contributed to their personal and professional growth • ECMH students further described a heightened sense of self-confidence and fulfillment
Yusoff et al. 2010 [39] (Malaysia)	Cross-sectional study	Mentors—select second year medical students—‘BigSibs’ Mentees—first year medical students *n* = 449 students; breakdown unspecified	**Recruitment of mentors**: Selection based on academic performance, ‘BigSib’ **Allocation of mentees to mentors**: Not specified **Contact logistics**: Monthly 2-h meetings, method of contact not specified **Training and orientation provided**: None **Activities organised**: Meetings, indoor games, workshops, treasure hunts, hiking, public speaking, picnics, outings **Duration of program**: Academic year	Conclusion of program	Validated questionnaire administered to mentors and mentees, with two domains measuring perception of the program in promoting campus life, mental health and wellbeing and confidence and respect of peers **Data analysis**: Chi-square goodness of fit test for categorical variables, Pearson chi-square to establish relationships and multiple logistical regression for individual effects of targets	• Majority of mentees felt as though the program did little in aiding communication between students and lecturers, and that the aims and objectives of the program were not made clear to them • Mentees (60%) felt that the program improved soft skills, professionalism • Less than half (45.9%) perceived the program as effective, but overall, it was perceived in a positive light • Program (from mentees perspective) was seen to aid in study skills, stress reduction, improve confidence and assist in adjustment to campus life
Focused programs						
Barker et al. 2012 [32] (UK)	Mixed methods study	Mentors—fifth year medical students (*n* = not specified) Mentees—first year medical students (*n* = not specified)	**Recruitment of mentors**: Volunteer **Allocation of mentees to mentors**: Random **Contact logistics**: Mentees expected to contact their mentor prior to the day **Training and orientation provided**: None **Activities organised**: Hospital orientation day (HOD) for early clinical exposure. Pre-visit planning sheet (mentees), orientation to the hospital environment, mentees to join fifth years in a teaching session **Duration of program**: One day	Immediately post intervention (survey) One-year post intervention (focus group)	One questionnaire and a focus group administered to mentees Non-validated questionnaire, consisting of questions regarding the organisation of the HOD. The answers were reported, and further, used to guide the focus groups the following year Focus groups (number of participants ranged from 4 to 5), semi-structured, digitally recorded and transcribed **Data analysis**: Not specified	• Mentees reported little to no fear regarding entering clinical placement, which was attributed to work experience, pre-visit contact and following the fifth year as key factors • Mentees found positive interactions with clinical staff and patients reassuring • Mentees saw the day as an insight into what their future in clinical medicine would look like and was associated with improved motivation • Mentees gained an understanding of the culture of the hospital and clinical learning and commented on the self-directed and autonomous learning styles of the fifth years • Mentees saw the day as valuable in facilitating discussions with clinical students Perception of the day was deeply affected by the ‘quality of the fifth year’. Characteristics that had made a quality mentor in this context included: ‘friendly’, ‘helpful’, ‘interested’, ‘proactive’ and ‘cheerful’
Choudhury et al. 2014 [28] (USA)	Cohort study	Mentors—fourth year medical students (*n* = not specified) Mentees—first year medical students (*n* = 77)	**Recruitment of mentors**: Volunteer **Allocation of mentees to mentors**: Random **Contact logistics**: Mentees joined Mentors at one of four student run clinics **Training and orientation provided**: None **Activities organised**: Interview skill practice, patient interactions, examination practice, case presenting, clinical note writing **Duration of program**: Duration of clinical placement; not specified	Immediately post intervention	Questionnaire (piloted and optimised 2012 prior to study initiation) administered to mentees, consisting of questions regarding frequency of volunteering, improvement in clinic-associated skills, and the role fourth year mentors played in improvement. General questions inquired about the fourth-year presence, role and impact on mentee comfort **Data analysis**: One-sample Wilcoxon sign-ranked median test and ordered logistic regression	• Mentees reported increased comfort with patients in clinic, and increased level of mentorship due to mentor presence • Mentees did believe the role played by fourth year mentors was different to the role played by attending physicians but found that fourth year presence enhanced mentee interactions at a comparable level • The perceived improvement was independent of which clinic the mentees attended, as well as the number of clinics volunteered at, frequency of volunteering or the number of mentors at the clinic • Logistic regression showed significant association between mentor presence and self-reported improvement in physical exam skills, and case presentation skills at one clinic site (however, no association with patient interviewing and note writing skills), but not at the other three sites
Lynch et al., 2022 [34] (US)	Mixed methods	Mentors—‘clinical-level students’ who had already taken the USMLE step 1 (M3, M4) *n* = 75 (breakdown unspecified) Mentees—pre-clinical students or M3s yet to sit the USMLE step 1 (M2, M3); *n* = 125 (breakdown unspecified)	**Recruitment of mentors**: Volunteers **Allocation of mentees to mentors**: Random; opt-out program **Contact logistics**: Mentors to contact mentees via email and explain program, offer frequency of meetings **Training and orientation provided**: Mentors attended a mandatory 1-h training session outlining the ‘Step Sibling’ program aims—told NOT to provide academic support **Activities organised**: Mentors encouraged to organise wellbeing checks monthly **Duration of program**: October 2020–March 2021 (beginning of year to USMLE Step 1 test)	Immediately post USMLE Step 1 exam	Questionnaire aimed at ‘elicit[ing] student reflections on the program’s effect on their stress levels and overall wellness and to seek feedback for future improvements to the program’ **Data analysis:** Response data evaluated for trends in agreement/disagreement Qualitative thematic analysis for comments	• 91% of mentees took exam after participating in program • 77% of mentees felt they were being contacted sufficiently by their mentors; 56% of mentees agreed that they found the program helpful; 53% of mentors agreed that they felt they were helpful • Majority of mentors (70%) and mentees (62%) agreed that ‘sharing perspective and experience was helpful’; sharing resources described as least helpful role of mentors • Most mentees and some mentors expressed desire to offer academic support. Some mentees reported being confused about the aims of the program • Majority of mentees ‘found the program helpful in decreasing stress’. A minority did not find peer mentors helpful, citing ‘already having existing mentors or supports in place.’ • Future program would involve information session for mentees to outline aims of program and the intention to provide wellbeing support only
Pinilla et al. 2015 [33] (Germany)	Mixed methods study	Mentors/mentees—First and second preclinical year medical students in Facebook groups for their respective year level First years *n* = 1149 Second years *n* = 1213	**Recruitment of mentors:** No formal recruitment of mentors, who were existing Facebook users present in year-level groups **Allocation of mentees to mentors:** To be accepted into the Facebook group by people already in the group **Contact logistics:** no formal contact organised; posts and comments as needed in group **Training and orientation provided:** None **Activities organised:** Posts and comments in two preclinical year Facebook groups **Duration of program**: 5 months	At the beginning of each semester, time periods directly correlating to critical written and oral exams, and between examination periods	Analysis of all posts and comments in the two preclinical year Facebook groups using a social constructivist perspective to identify emerging peer-mentoring themes that are relevant for undergraduate medical students and reflect social norms, values and needs of medical students in this context. Anchoring examples were defined for each peer-mentoring subcategory and the final coding scheme was applied to three critical weeks of each preclinical year with particularly high or low posting frequency, as well as at the time of high-stakes exams Frequency of peer-mentoring related elements in posts and comments was reported Two focus groups with medical students enrolled in different semesters were conducted	• The preclinical year 1 (PCY1) Facebook group (*n* = 1149) and preclinical year 2 (PCY2) Facebook group (*n* = 1213) had significant number of individuals being members of both Facebook groups, with enrolled PCY1 and PCY2 students being 950 and 966 respectively • Most common categories for PCY1: social activities, study related topics and resources. Most common categories for PCY2: social activities, study related topics and knowledge/skills • More complex peer-mentoring elements such as empowering and fostering personal development seem to be underrepresented in these groups From the focus groups, 3 major themes emerged: similarity in experience and rank, pool of skills/experiences/resources within a group and empowerment and emotional support
Prunuske et al. 2019 [16] (USA)	Qualitative study	Mentors—second year Native American and Alaskan Native medical students (*n* = 9) Mentees—First year Native American and Alaskan Native medical students (*n* = 40)	**Recruitment of mentors**: Selected based on interpersonal skills and academic performance. Mentors were paid an hourly salary **Allocation of mentees to mentors**: 1 mentor to 4.5 mentees, allocation method not specified **Contact logistics**: Daily interaction via activities coordinated by program organisers **Training and orientation provided**: None **Activities organised**: Clinical skills, problem-based learning, microbiology labs and faculty lectures **Duration of program**: 4 weeks for each year however program ran for 5 years with 5 different cohorts	6 months after program conclusion	Semi-structured interview administered to mentors, comprising 19 questions regarding what they gained, what could be improved and what sort of guidance was provided to the mentees **Data analysis**: Not specified	• Role was described by mentors as beneficial in several ways: emotional/social support, academic support and give an insider view of medical experience • Social congruence was a common theme, under comments such as ‘you remember what it feels like’ etc • Mentors noted that small cohorts and frequent meetups strengthened the relationship between mentee and mentor • Mentors also found the experience beneficial to consolidating their knowledge and skills, as well as reinforcing a commitment to rural First Nations health

^*^Two reports of the same program

Peer mentoring programs could broadly be divided into two types. Fifteen studies (two describing the same program) were ‘longitudinal’ programs, aimed at establishing mentor–mentee relationships to offer ongoing support over a longer period, such as an entire academic year. The remaining five studies were more ‘focused’—generally shorter-term programs with the intent of providing mentorship during significant moments for mentees, such as transition to clinical placement, medical school orientation or exam periods. Accordingly, where programs were named, these names generally reflected the goals—‘BigSibs’ [39] and ‘Mentors in Medicine’ [29] for longitudinal programs, compared to ‘Step Sibling’ [34], which focused on providing (non-academic) support for the USMLE Step 1 exam.

In terms of outcome measurements, 17 studies used quantitative tools, mostly self-developed (piloted or validated in four studies [28, 29, 31, 39]). Three studies used standardised and validated tools: Mentorship Effectiveness Scale [27], DREEM [37] and LCQ, W-BNS and PCS [26]. Assessment timepoints were short-term (start and end of the programs). Only one study [31] assessed additional longitudinal outcomes (12 months post-program). Results were analysed for statistical significance in 12 studies [18, 19, 26–30, 34, 36–39].

Outcomes for longitudinal and focused programs respectively are presented in the following section, outlining benefits, elements for program success and challenges.

### Longitudinal Programs

#### Study Characteristics

Of the 20 included studies, 15 described longitudinal near-peer programs. Thirteen spanned the duration of the academic year, whilst one was semester-long (12 weeks) [31] and another, 4 years long [36]. Two programs were directed at preclinical students who were transitioning to clinical placement [30, 31]. The remaining 13 studies included first or second year students as mentees, and two involved students across all year levels [29, 36].

Mentors were typically senior students in their second (five programs) or fourth year (two programs), or students from multiple year levels (six programs). Two programs also involved doctors as mentors [30, 36]. One program paired mentees with either mentors in the same year level as the mentee, or two academic years senior [30]. The other program paired student and faculty mentors [18]. The program initially commenced with faculty mentors only, but added student mentors following feedback that mentees were reluctant to meet their mentors. Subsequently, students met more often with both peer and faculty mentors [18]. In contrast, a study by Yang et al. compared the experiences of mentees who had received mentoring from peer mentors to those who had received mentoring on a one-on-one basis with a physician; there were no reported barriers to engagement; however, there was a notable component of teaching in this mentoring program, and attendance was mandated [36].

#### Benefits

Studies assessed the impact of their programs on either the mentors, mentees or both. Seven studies [14, 18, 26, 29, 30, 35, 39] assessed outcomes measures for both, whilst three [13, 19, 27] assessed mentee outcomes and three [17, 31, 36] mentor outcomes. Of the seven studies which assessed outcomes for both, all studies reported positive outcomes in confidence, perceived learning environments and psychosocial wellbeing for both mentees and mentors. Where peer mentors or physician mentors were compared, there was no difference in reported mentee benefits [36].

Positive outcomes for mentees were academic development, psychosocial wellbeing and communication skills. Five studies [14, 29, 30, 35, 39] reported mentees felt more confident with their academic skills due to skills gained from mentors, such as academic planning [29] and study techniques [35]. Mentees also perceived the learning environment more positively [13, 26, 31, 36, 37] and reported improvement of social, interpersonal [30, 35] and clinical communication skills [14]. Two studies [29, 30] found improved psychosocial and emotional peer support for mentees, and one study [37] showed mentees were more likely to feel they had a good support system in times of stress. The provision of a ‘safe space’ for mentees was highlighted in three studies [13, 29, 35]. Another study emphasised that mentees felt more competent and autonomous as learners in the learning climate fostered by near-peer mentors [26].

Mentor outcomes were academic and interpersonal skills gained from mentoring, and overall satisfaction with mentoring. Mentors perceived improvement in professional skills, such as responsibility, leadership and communication [18, 30, 31, 35, 36, 38]; moreover, they felt satisfied with their experience [14, 36, 38, 39], and more confident when learning and teaching [26].

### Elements for Peer Mentoring Program Success

#### Expectations of Regular Contact

Although program structure varied, all expected regular contact, with seven programs [14, 29–31, 35, 37, 39] outlining minimum requirements for mentor/mentee contact frequency, ranging from twice weekly [14], to weekly [31, 35, 37], and at least monthly [29, 39]. The only program where contact was mandated was the study by Yang et al. [36]. Most studies did not report frequency of contact and it is unclear how often mentors and mentees met. Two of the studies found that synchronising meeting times was a common barrier; however, this was less of a barrier with peer mentors than with faculty mentors [18, 19].

#### Face-to-Face Communication

Students reported using a variety of methods including face-to-face [13, 26, 29, 30, 35, 37, 39], text messages and email, with face-to face reported as most beneficial in one study [35] and most utilised in another [30].

#### Clear Agendas

Nine programs encouraged student-driven agendas for mentor–mentee meetings whilst five programs were more prescriptive, focused around clinical topics [13, 14, 26, 31, 36], or non-clinical academic topics such as professionalism and social supports [29]. One study further facilitated the program though the organisation of social activities [39].

#### Orientation and Training for Mentors

Orientation and training was provided for mentors in seven studies [14, 17, 27, 30, 31, 35, 37]. This generally involved outlining the purpose of mentoring and program expectations [30, 37], communication, leadership and/or interpersonal skill development [30, 35, 37]. These ranged from one-off sessions [14, 27, 30] to more intensive 2-day [31], 3-day [37] or multi-session training [17].

Overall, training was viewed favourably. Mentors reported an increased ability to provide constructive feedback post orientation [14], and that training improved their communication, leadership and interpersonal skills, in both personal and professional domains [31]. In one study with no orientation or training, mentors reported that they would have benefited from clearer aims, objectives and guidelines [39], and further, mentors in one study reported that more than one session was required [30].

### Challenges

The balance between training and overload of mentors was important, as mentoring and training was on a voluntary basis [27, 30]. Matching mentees with mentors intentionally was received positively. However, the authors of one study noted that this may be more logistically intensive, and felt more evidence was needed before such measures were implemented [27].

### Focused Programs

#### Study Characteristics

Five studies described focused mentoring programs with targeted aims [16, 28, 32–34]. There was more heterogeneity across these studies in terms of program structure and setting and aims. The shortest of these was 1 day [32], targeted at hospital orientation, and the longest 6 months, targeted at support for the USMLE Step 1 exam [34]. One study provided a single training session for mentors [34].

#### Benefits

For programs with a clinical focus, outcomes were measured through self-developed questionnaires, which found positive findings in increased comfort, reduced fear and improved understanding of self-directed learning styles or clinical skills [16, 28, 32]. However, these questionnaires were not externally validated. The First Nations peer mentoring program found that the program reinforced the commitment of mentors to rural First Nations health [16].

The ‘Step Sibling’ program [34] also reported positive overall findings, with general agreement from both sides that the sharing of perspectives and experiences had been helpful. Mentees reported that the program had decreased their stress. It was also reported that mentees felt the least useful role of mentors was in sharing resources.

### Elements for Peer Mentoring Program Success

#### Clear Focus

Of the five programs, three [16, 28, 32] had a clinical focus, designed to familiarise mentees to a clinical environment and the teaching activities that occurred in that setting. Of these, one was developed specifically for First Nations students [16]. In contrast, the ‘Step Sibling’ program [34] was more exam-focused, specifically designed to offer psychosocial support to students undertaking the USMLE step one exam.

#### Matching of Mentors and Mentees Based on Additional Shared Characteristics

This was an important factor especially for under-represented groups [16].

#### Remuneration

The program for First Nations students reported that mentors were paid a salary [16].

### Challenges

Interestingly, one study utilised social media as a means for mentoring for large groups of students [33]. Social interactions within a year-level were observed in two large first (*n* = 1149) and second (*n* = 1213) year-level Facebook groups. Thematic analysis of posts identified peer-mentoring themes. However, whilst students appreciated the experience and pool of resources, complex peer-mentoring elements such as empowerment and fostering personal development were absent.

## Discussion

Our scoping review included 20 peer mentoring studies where both mentors and mentees were medical students. Of these, most (15) were longitudinal programs, aimed at first year students or the pre-clinical to clinical transition. All programs reported academic (development of clinical confidence, skills in patient care, procedural skills, medical knowledge, interviewing and examination skills) and non-academic (confidence, social support, peer relationships and communication skills) benefits for both mentors and mentees. Further, many studies identified elements for success such as orientation and training for mentors and challenges such as avoidance overload of mentors and logistics.

These findings are consistent with those of previous reviews [15, 21–23], and affirm the role of ‘social congruence’. The included studies found that mentees highlighted the ability for mentors to empathise with mentees as an important element of effective peer mentoring [13, 16, 19, 20, 26, 29, 34, 36]. They also felt that social congruence allowed the establishment of a ‘safe space’ in a peer mentoring group [13, 26, 29, 35]. Although not the primary aim of peer mentoring, it was notable that mentees developed academic and professional skills likely as a result of role modelling from mentors [15].

This review identified several elements that were important for a successful program. Firstly, a number of included studies highlighted the importance of matching mentors and mentees based on additional shared characteristics such as clinical experiences or demographics [14, 16, 17, 27, 30, 37, 38]. These shared traits could facilitate the initiation of a mentoring relationship. A recent systematic review suggested that sharing of specific socio-demographics may be particularly important for more vulnerable mentees such as underrepresented groups [24]. Secondly, an important element of program success was the orientation and training provided to mentors which clarified their role and the objectives of the program [14, 17, 27, 30, 31, 34, 35, 37] and this was reinforced as a recommendation by programs where this was lacking [18, 32, 39]. Orientation was also important for mentees [30, 34, 39]. Interestingly, two studies reported that training was not necessary, given students had spent time as a mentee prior to being a mentor [16, 36]. However, these exceptions would require further evaluation. A related consideration was the balance between training and overload of mentors as ‘extensive, uncompensated training (could) discourage volunteer mentors’ [27]. As part of the training, consideration could be given to ensuring mentors understand the role of greater support systems, especially since perceived independence of the program from the medical school was seen as a bonus for mentees and mentors [35]. This would mitigate concerns which have been raised where the independence of peer mentoring programs from faculty staff could potentially result in the loss of other, formalised support systems [22]. Bias awareness training on diversity and inclusion could also be considered for a more inclusive environment [40].

Other elements to consider for program success raised by a small number of included studies included (1) the possibility of protected time allocation for peer mentoring [23]; students nevertheless reported their relatively flexible schedules made meeting peer mentors far easier than faculty mentors [18, 19]; (2) face-to-face meetings where possible [30, 35]; (3) small group sizes [16, 21] given large social media platforms did not allow for the more complex elements of peer mentoring [33]; and (4) remuneration [16] especially for disadvantaged groups which could allow for improved equity. Interestingly, common resource limitations such as cost and sustainability were not raised by the studies as elements for success.

Limitations of this study included the challenges of developing a search strategy in the absence of ‘mentoring’ as a MeSH term and the heterogeneous use of terminology (such as ‘buddy’) to describe mentoring programs. Several keywords were used in the search strategy for comprehensiveness; however, it is possible these did not capture all available studies. It is also possible that peer mentoring programs were missed in the exclusion of peer-assisted learning studies; however, there was a low threshold for full text reviews to be conducted when uncertain. Further, restrictions to English language only and to the listed databases meant that it is possible that studies in other languages and/or other databases could have been missed. All included studies had significant methodological weaknesses, such as lack of randomisation, blinding and control groups. There was also heterogeneity of the study designs and outcome measures which made comparisons challenging and all included studies reported at least one positive outcome, raising the possibility of publication bias although a grey literature search was conducted.

This scoping review has highlighted significant gaps in the current literature, and hence, further research should consider the following:

- Using robust methodology with validated outcome measures and performing statistical analysis for significant differences.
- Comparing different types of interventions (given the ethical difficulties of having controls).
- Including delayed outcome measures to determine longitudinal outcomes.

Based on the findings of this review, educators should consider the following recommendations when designing peer mentoring programs:

- Clearly specified aims, outcomes and roles for mentors and mentees.
- Training and orientation for mentors.
- Longitudinal programs provide support for students as their experiences change throughout their degree. Multi-year programs enable a deeper development of relationships which may increase participation and retention.
- Shorter, focused programs specifically for high-stress points, such as transition points or examination times.

## Conclusion

Our review found that peer mentoring programs where medical students are mentored by medical students provide benefits, including improving psychosocial wellbeing and academic development. Challenges in development and implementations included workload concerns for mentors, logistical concerns for face-to-face programs and financial limitations.

Key elements which optimise delivery of these programs include orientation and training for mentors and a clear outline of roles for both. Medical educators could consider the implementation of these programs as part of their medical school curricula.

## Data Availability

Data sharing is not applicable to this review as no datasets were generated or analysed during the current study. All databases used in this study were publicly accessible at the time of submission.

## Appendix. Search Strategy

### PubMed

((((((((medical student[MeSH Terms]) OR (medical students[MeSH Terms])) OR (students, medical[MeSH Terms])) OR (mentor[MeSH Terms])) OR (mentors[MeSH Terms])) AND (((((group, peer[MeSH Terms]) OR (peer mentor*)) OR (peer-mentor*)) OR (near-peer*)) OR (near peer*))) AND (medical education[MeSH Terms])) NOT (((((peer* assist* learn*) OR (nurs*)) OR (dental*)) OR (residen*)) OR (intern*))) AND (English[Language]).

### ERIC

((medical OR medicine) N2 student*) AND (peer mentor* OR near-peer* OR near peer*).

### EMBASE

- 1. medic* student*.mp.
- 2. (peer group or near-peer* or near peer*).mp.
- 3. (mentor* or support).mp.
- 4. (peer* adj3 mentor*).mp.
- 5. 1 and 2 and 3.
- 6. 1 and 4.
- 7. 5 or 6.
- 8. limit 7 to English.

### Web of Science

- 1. (ALL = (medical student*)) OR ALL = (medicine student*).
- 2. ((ALL = (near-peer*)) OR ALL = (peer mentor*)) AND ALL = (medical education).
- 3. ((((ALL = (nurs*)) OR ALL = (dental)) OR ALL = (intern*)) OR ALL = (residen*)).
- ((#1) AND #2) NOT #3

### Scopus

(medic* PRE/0 student* AND peer PRE/1 mentor*) OR ( near-peer* OR near PRE/1 peer* AND medic* PRE/1 student) AND NOT ( nurs* OR dental OR intern* OR residen*).

### WorldWideScience

Medic* student* AND (peer mentor* OR near-peer*) AND (medical education) NOT (nurs* OR dental* OR intern* OR residen*).

### British Library

(medic* student* and mentor) OR (near-peer* and medic* education) NOT (nurs* or dental* or paramed* or intern.
